# Novel ANOVA-Statistic-Reduced Deep Fully Connected Neural Network for the Damage Grade Prediction of Post-Earthquake Buildings

**DOI:** 10.3390/s23146439

**Published:** 2023-07-16

**Authors:** K. R. Sri Preethaa, Shyamala Devi Munisamy, Aruna Rajendran, Akila Muthuramalingam, Yuvaraj Natarajan, Ahmed Abdi Yusuf Ali

**Affiliations:** 1 Department of Robot and Smart System Engineering, Kyungpook National University, 80, Daehak-ro, Buk-gu, Daegu 41566, Republic of Korea; k.r.sripreethaa@kpriet.ac.in; 2 Department of Computer Science and Engineering, KPR Institute of Engineering and Technology, Coimbatore 641407, India; 3 Vel Tech Rangarajan Dr. Sagunthala R&D Institute of Science and Technology, Chennai 600062, India; shyamaladevim@veltech.edu.in (S.D.M.); drraruna@veltech.edu.in (A.R.); 4 Department of Electrical Engineering, University of Johannesburg, Johannesburg 2006, South Africa

**Keywords:** ANOVA, activation, classifier, PCA, gradient descent, regularization, DFCNN

## Abstract

Earthquakes are cataclysmic events that can harm structures and human existence. The estimation of seismic damage to buildings remains a challenging task due to several environmental uncertainties. The damage grade categorization of a building takes a significant amount of time and work. The early analysis of the damage rate of concrete building structures is essential for addressing the need to repair and avoid accidents. With this motivation, an ANOVA-Statistic-Reduced Deep Fully Connected Neural Network (ASR-DFCNN) model is proposed that can grade damages accurately by considering significant damage features. A dataset containing 26 attributes from 762,106 damaged buildings was used for the model building. This work focused on analyzing the importance of feature selection and enhancing the accuracy of damage grade categorization. Initially, a dataset without primary feature selection was utilized for damage grade categorization using various machine learning (ML) classifiers, and the performance was recorded. Secondly, ANOVA was applied to the original dataset to eliminate the insignificant attributes for determining the damage grade. The selected features were subjected to 10-component principal component analysis (PCA) to scrutinize the top-ten-ranked significant features that contributed to grading the building damage. The 10-component ANOVA PCA-reduced (ASR) dataset was applied to the classifiers for damage grade prediction. The results showed that the Bagging classifier with the reduced dataset produced the greatest accuracy of 83% among all the classifiers considering an 80:20 ratio of data for the training and testing phases. To enhance the performance of prediction, a deep fully connected convolutional neural network (DFCNN) was implemented with a reduced dataset (ASR). The proposed ASR-DFCNN model was designed with the sequential keras model with four dense layers, with the first three dense layers fitted with the ReLU activation function and the final dense layer fitted with a tanh activation function with a dropout of 0.2. The ASR-DFCNN model was compiled with a NADAM optimizer with the weight decay of L2 regularization. The damage grade categorization performance of the ASR-DFCNN model was compared with that of other ML classifiers using precision, recall, F-Scores, and accuracy values. From the results, it is evident that the ASR-DFCNN model performance was better, with 98% accuracy.

## 1. Introduction

Earthquakes are calamitous events that can harm structures and human existence. The seriousness of earthquake-actuated structure damage relies upon factors like size, distance from the focal point, land conditions, and seismic structure execution. The rapid assessment of the distribution pattern and severity of damage to buildings is critical for post-event emergency response and recovery [1]. According to Samuel Roeslin et al., earthquake information from the seismic tremor perception division shows that small seismic tremors often happen in Northern Thailand. On 5 May 2014, there was a 6.3 seismic tremor in Chiang Rai Province [2]. The strike was a tremendous recorded seismic tremor in Thailand, damaging structures in a vast region. The time of a potential earthquake is unpredictable. Consequently, seismic danger evaluation is vital for readiness for proper tremor relief exercises. An exhibition-based quake-designing evaluation technique was created to give an essential dynamic seismic arrangement. According to the suggestions of Rao, M.V.V. et al., a detailed nonlinear relocation-based strategy is a complex computation for improving a structure’s seismic presentation. Machine learning strategies are computationally requested to evaluate enormous topographical regions [3]. Tianyu Ci et al. stated that, after an earthquake, it is crucial to assess the level of endangerment sustained by buildings so that people can avoid being in unsafe structures. The post-earthquake structure of a building must be restored with a proper analysis of the damage. Appropriate technology must be utilized to categorize the damage to the building facilities. Building damage is visually observed and recorded. Manual assessment and categorization are labor-intensive and time-consuming and may take place for months after the disaster. Machine learning (ML) and deep learning (DL) techniques could be applied as a tool for the faster assessment of damage and early restoration, thus preventing loss of life and property [4].

The main objective of this research was to investigate how much the performance of a deep, fully connected neural network could be improved by tuning the input features and parameters of the model compilation. For this purpose, this research is explained as follows. Section 2 outlines the past studies related to damage grade classification using ML and DL techniques. Section 3 describes the research workflow, the steps to frame the proposed ASR-DCNN model, and the module workflow with the architecture. Section 4 explains the implementation steps of data preprocessing and model training. Section 5 discusses the results of experimentation at various stages of development. Section 6 concludes by summarizing the findings and the future scope for enhancements.

## 2. Literature Review

In recent years, ML and DL algorithms have been widely used in various domains. Specifically, in applications in which rapid and accurate analysis is required for large data, ML and DL methods are necessary. ML and DL algorithms significantly improve automation and accuracy enhancement in structural analysis. Based on the severity of the earthquake, there are five grades of damage to building structures. The damage level of the buildings can be assessed by the data gathered using regression methods [5]. Xiao-Wei Zheng et al. stated that ML algorithms are instrumental when the damage-sensitive features obtained from the seismic response are influenced by operation, environmental variability, and damage. ML techniques generalize the normal structural behavior to detect deviations from the baseline period associated with destruction. To assess the risk of damage, high-rise building seismic data can be studied [6,7]. Although the researchers have worked on incorporating various types of ML and DL to assess the damage risk of earthquake-affected buildings, there is a vast need for a new methodology that effectively assesses the damage grade of a facility by considering all the parameters of the building damage details, according to the analysis of Kim, B et al. [8]. According to Mangalathu S. et al., after an earthquake, it is crucial to assess the level of endangerment sustained by the buildings so that people can avoid being in unsafe structures after the calamity. The potential advantage of using ML methods, such as discriminant analysis, k-nearest neighbors, decision trees, and random forests, is that they support the faster prediction of earthquake-induced damage to structures. Using a subset of Napa earthquake data, a predictive model was developed and assessed using various ML methods, among which random forest achieved 66% accuracy in predicting the building damage [9]. Based on the analysis of Chaparala A et al., building structural integrity can be monitored using hybrid machine learning techniques (HMLT). HMLT integrate improved classifiers such as support vector machines (SVM) and artificial neural networks (ANN), to predict the strength level of buildings. The HMLT efficacy yielded a 91% F1-score and 92% accuracy [10]. Saengtabtim K et al. accessed the calamities of the great East Japan earthquake and tsunami of 2011. They initially checked whether the maximal values of the flow depth and flow velocity matched their critical values, and then determined a combination of the parameters that accurately estimated the structural damage level. Implementing the decision tree algorithm using critical flow depth and maximum flow velocity produced the highest accuracy for determining the degree of building damage [11].

Kim B. et al. stated that combining convolutional neural network (CNN) with an ordinal regression model efficiently assessed the damages in buildings caused by earthquakes [12]. Ci, T. et al. developed a deep learning model for examining the stages of building damages that produced 93.95 accuracy with hyperparameter tuning [4]. The performance of the CNN-based model in transfer learning was promising, specifically in the geographical transferability of the trained network to imagery acquired in different locations, spatial resolutions, and platforms using the heterogeneous and substantial datasets gathered by Nex, F et al. The results demonstrate that the composition of training samples used in the network influences metrics quality. The pre-trained networks, optimized for satellite, airborne, and unmanned aerial vehicle (UAV) image spatial resolutions and viewing angles, are readily available to the scientific community to encourage their broader application [13,14]. According to the findings of Ahadzadeh S. et al., the cost and time of disaster management are significant. Also, the presence of the data acquired before the event is essential to evaluate the data after an earthquake. Since earthquake behavior is typically nonlinear, neural networks can be a suitable approach [15].

P Debnath, P et al. found that, in geoscience, ML classifier algorithms could categorize the earthquake as fatal, mild, or moderate from the range of seismic magnitude. The model implemented seven machine learning classifiers using six datasets collected from India and neighboring regions [16]. Artificial-intelligence-based (AI) solutions were frequently applied to solve civil engineering problems involving modeling and optimizing large structure systems and enormous computing resources. AI applications in civil engineering were developed using chaos theory, cuckoo search, firefly algorithm, knowledge-based engineering, evolutionary algorithms, simulated annealing, neural nets, fuzzy systems, optimization, reasoning, categorization, and learning techniques [17].

The long short-term memory (LSTM) DL-based algorithm was applied to categorize the building damage based on the textual descriptions documented after the earthquake in 2014 in South Napa, California [18]. The investigation of Kumari V. et al. shows that applying AI methods to civil engineering applications has produced encouraging outcomes and minimized human interaction, including uncertainty and biased judgment. A dataset containing various earthquakes explored many well-known nonparametric techniques toward rapid visual screening (RVS). The methodology also offers the potential to evaluate the sensitivity of the buildings considering their significance and exposure [19]. Alvanitopoulos, P.F. et al. state that engineers must assess the safety of existing structure facilities and choose the appropriate course of action after an earthquake. ANN and neuro-fuzzy systems were applied to automatically classify building damages using 20 seismic parameters that characterize the basic information included in accelerograms [20]. 

Lazaridis, P.C. et al. studied structural damages using actual and synthetic ground shaking sequences and reported two total damage indices. The comparison analysis yielded the most effective damage index and the best machine learning algorithm for predicting how a reinforced concrete building would respond structurally to a single or series of seismic occurrences [21]. Morfidis K. et al. suggested that ANN-based methods for the seismic vulnerability assessment of structures could be used as alternatives to existing well-established methodologies. ANNs were not widely accepted as computational methods for predicting the seismic damage level of structures, as most civil engineers researching methods for assessing a structure’s seismic vulnerability needed prior knowledge regarding the capabilities and applications of ANN-based methods [22]. Rashid, M. et al. discovered that the destruction to internal structures was determined for each intensity level and integrated throughout the structure with the cost of necessary repairs to determine the structure repair cost ratio (RCR). The seismic vulnerability curves can be used to estimate the economic loss (direct repairability cost) of SMRF structures and the structural RCR correlated with the seismic intensity [23].

Rao, M.V.V. et al. analyzed the state of the structure and determined its potential risk was two critical goals of structural strength-monitoring frameworks. Investigating, identifying, and characterizing risk in complicated structures is crucial to additional strength checking. The capacities are explored as scaled variations of a fundamental Gaussian hypothesis task and a vocabulary of time-recurrence movement. Characterization is then completed by synchronizing the eliminated damaged components with the time frequency. Signals collected from sensors are disintegrated into direct blends of minimal Gaussian capabilities using the coordinated significance decay computation. The balanced scratch-off and high-pass sifting procedures are sufficiently combined to address challenges in numerical reconciliation. As opposed to earlier numerical integrators, the combination’s accuracy was improved. In contrast to fuzzy set methods, the rough set analysis uses internal knowledge and does not assume any prior models [24].

The structural building health damage monitoring system (SBHDMS) is a powerful technology for predicting the health of civil building structures. Buildings in SBHDMS have undergone unusual alterations in terms of damage levels. Earthquakes, floods, and cyclones are natural disasters that have an extraordinary impact on structures. The sensors record the vibration data and are used to alter the construction of the building in the event of a natural disaster. The peculiar variations were examined according to the vibration data. The RAS approach forecasts the vibration data levels recorded by the sensors in the damaged structure [25].

Gemma Cremen et al. claimed that the early detection of earthquakes reduces the risk factor and environmental hazards. Earthquake early warning (EEW) systems make real-time information about active earthquakes available, allowing people in far-off communities, governments, companies, and other settings to take prompt action to reduce the likelihood of harm or loss before the earthquake-induced ground shaking reaches them. The limitation of the existing methods is the need for engineering-related metrics to make early detection decisions [26]. Natt, L. et al. showed that the outcomes of significant damage analysis parameters provide engineers, architects, and construction and disaster recovery management personnel with a practical understanding of the degree of damage experienced by buildings [27]. According to Chaparala A. et al., performing feature reduction or choosing features from the dataset is crucial before classifying data to ensure accuracy. Examining the dataset using the rough set theory helped to streamline the classification process by reducing the complexity of the feature selection procedure. Rough set theory analyzes the essential parameters without added data [28]. In order to forecast the flexural strength of ultra-high-strength concrete, Wang et al. [29] examined different supervised ML techniques such as decision tree bagging, decision tree gradient boosting, decision tree AdaBoost, and decision tree XG boost. The findings proved that DT bagging is the method that most closely approximates experimental outcomes. In order to show how seismically vulnerable existing structures are, Ruggieri et al. [30] published a vulnerability study utilizing machine learning (VULMA). The procedure comprised four modules that each offer specific and specialized services. Street VULMA begins by gathering raw data. Data VULMA offers a way to classify and store data. Bi VULMA rates the pictures, looks at them, and calculates the vulnerability index after using the gathered data to train multiple machine learning models for picture classification. The five most typical flaws in RC bridges may be classified and detected using convolutional neural network (CNN)-based Xception and Vanilla models for the picture categorization procedure. The two models were developed, tested, and compared using the concrete fault bridge images (CODEBRIM) dataset for multi-class and multi-target image classification. The results demonstrated the possible use of the Xception and Vanilla models to classify defects in concrete bridges and the superiority of the Xception model in highly accurate defect classification [31].

The literature outlined that varied methodologies were used to study the damage grade caused by earthquakes. Though ML and DL technologies can be utilized to assess the damage risk of earthquake-affected buildings, several challenges exist during implementation. The research challenges lie in considering the recorded dataset’s significant features, encoding the feature values, outlier analysis, sampling the target feature, and freezing the number of hidden layers with the appropriate activation function and optimizers. The present work focuses on building an efficient neural-network-based model with a competent dataset. For this purpose, varied techniques such as the ANOVA test and 10-component PCA methods are utilized.

## 3. Research Methodology

**Figure 1:**
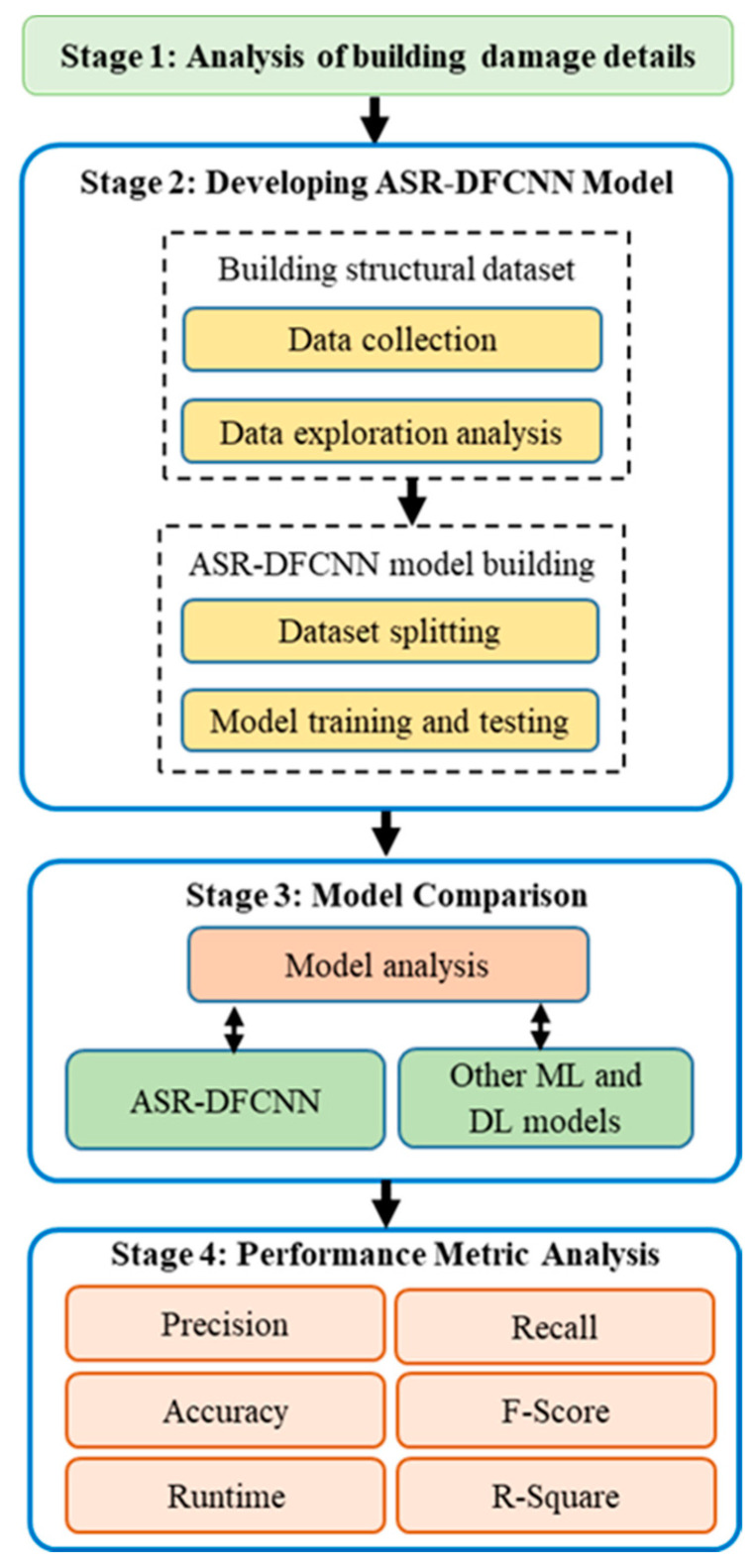
Research methodology of the proposed ASR-DFCNN system.

The proposed work aims to design an effective machine-learning-based model for categorizing post-earthquake building damages. The workflow of the proposed method is divided into four phases, as depicted in Figure 1. The first stage includes collecting related earthquake-affected building damage data from open-source repositories. Stage two involves data analysis, preprocessing, and constructing the ASR-DCNN framework for implementation. The exploratory data analysis visualizes the statistics and distribution of damage grade concerning the plinth area, foundation type, other floor types, ground floor type, land surface type, position, and roof type. During data preprocessing, missing data are imputed, and feature scaling, categorical encoding, and normalization are conducted. Damage grade classification using existing classifiers was applied to the imputed data by splitting the data into 60:40, 70:30, and 80:20. Then, the proposed ASR-DFCNN model was built by analyzing the performance of the cross-validation and ANOVA results. During stage three, the dataset was fitted to the proposed ASR-DFCNN model and validated for efficiency in predicting the damage grade of the building by comparing it with existing classifiers. At stage four, the proposed model was evaluated using performance metrics such as precision, accuracy, recall, F-score, and R-squared error.

### 3.1. Development of ASR-DFCNN Framework

**Figure 2:**
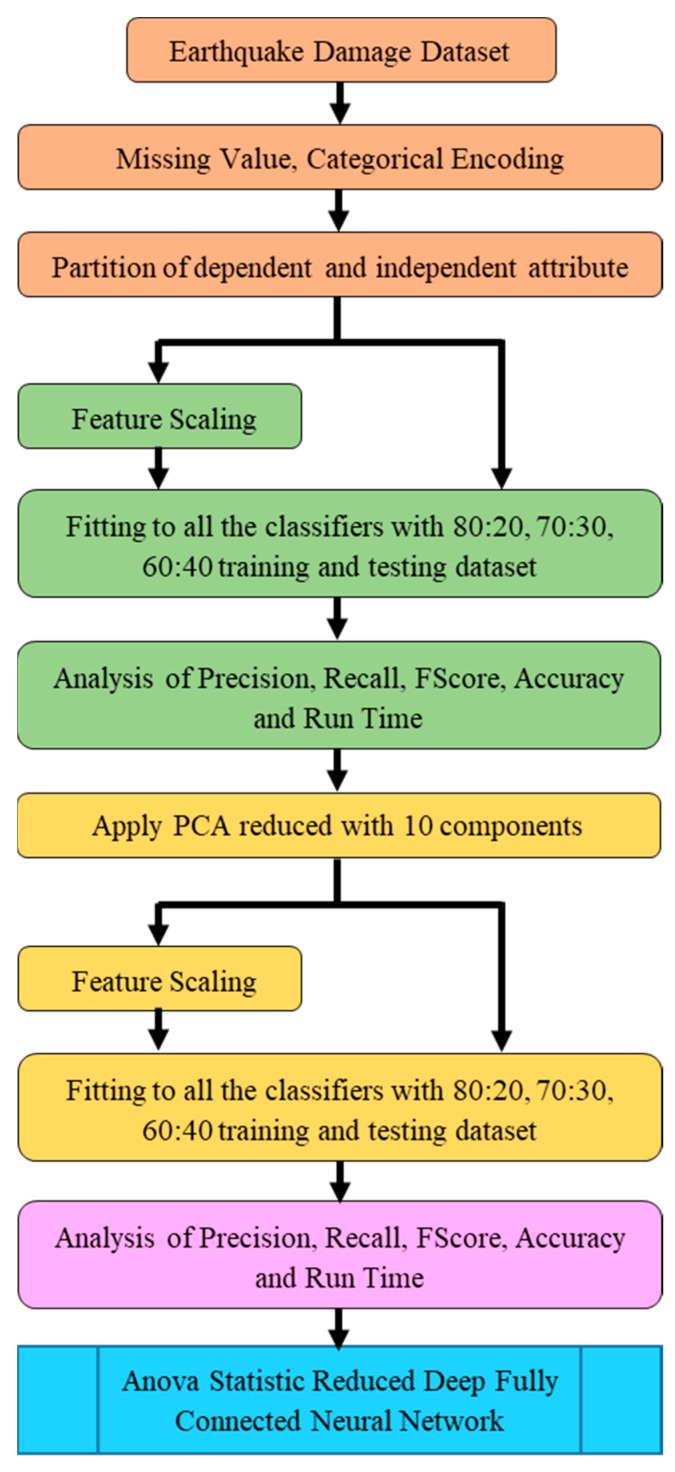
ASR-DFCNN model development framework.

The development steps of the proposed ASR-DFCNN framework model are depicted in Figure 2. The earthquake dataset collected from the Kaggle repository was preprocessed for missing values, and categorical encoding was conducted to generate the normalized dataset. The processed dataset was segregated into 25 independent variables and ‘damage_grade’ as the dependent variable. The dataset was then split for training and testing in the 80:20, 70:30, and 60:40 ratios, and then fitted with various existing ML classifiers. The performance of the classifiers was analyzed using precision, recall, accuracy, F-score, and run time. The results showed that classification with the classifiers exhibited better performance with a data split ratio of 80:20. The ANOVA test was applied to the preprocessed dataset to find the significance of the input variables in deciding the damage grade. The insignificant attributes were identified and removed from the dataset. Further, the most significant features that decide the damage grade were extracted from the dataset using a 10-component PCA model. After the ANOVA and 10-component PCA methods, the reduced dataset was used to create a deep, fully connected neural network for predicting building damage grade.

**Figure 3:**
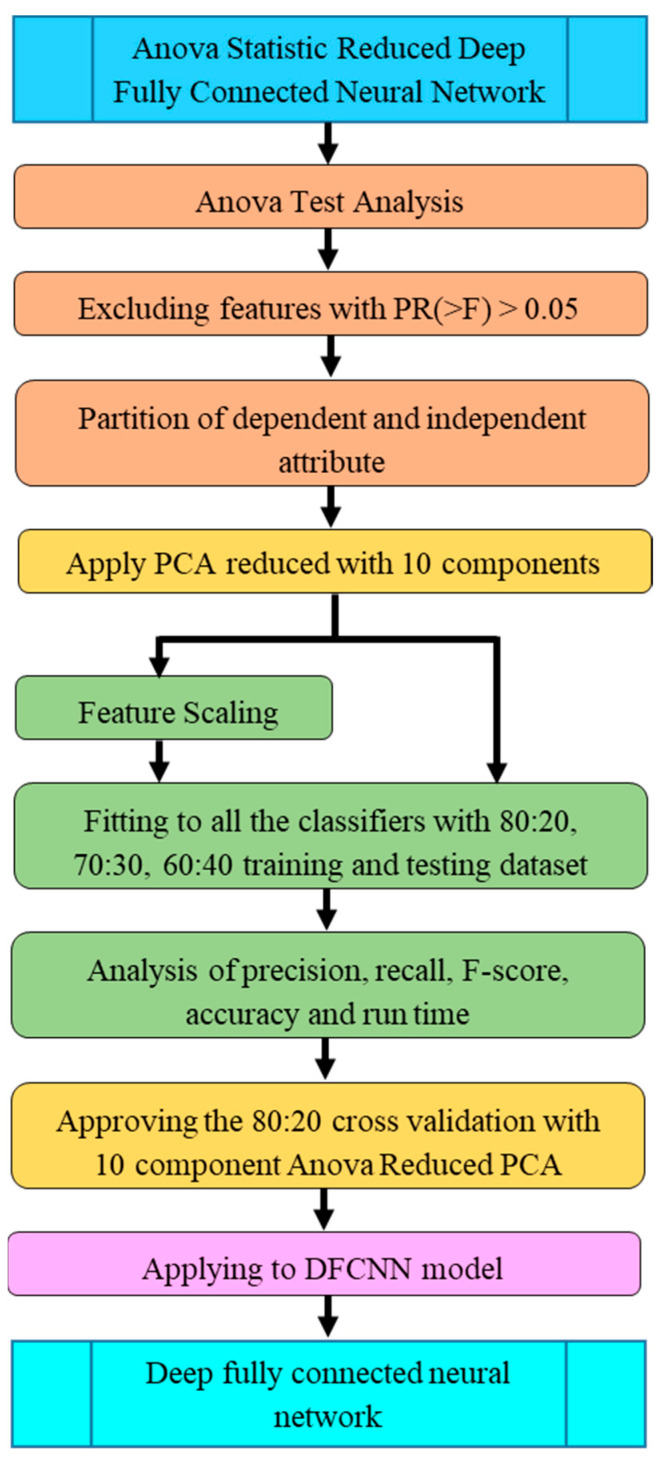
ASR-DFCNN module flow.

The module workflow of the ANOVA-statistic-reduced deep fully connected neural network is shown in Figure 3. The dataset with the entire 26 features was applied to all the classifiers before and after component scaling of the data to grade the damage. The performance was analyzed by cross-sectioning the training and testing data with 80:20, 70:30, and 60:40. The ANOVA test was applied to the dataset to identify the features that did not contribute to the damage grade prediction. Based on the results, insignificant variables were removed. Using a 10-component PCA method, the dataset was further reduced to 10 necessary variables to estimate the damage grade. The resultant dataset was applied to all the classifiers to grade the damage. The performance was analyzed in the presence and absence of component scaling at every step of dataset reduction, and the best-fit dataset was used for the building of the proposed model.

The ASR-DFCNN module initiated an ANOVA test that eliminated the insignificant variables that produced values below the threshold. Features selected through the 10-component PCA-reduced dataset were applied with all the classifiers to analyze the performance. The analysis showed that the DCNN model and 80:20 data splitting were approved to construct the proposed ASR-DFCNN model.

### 3.2. Architecture of ASR-DFCNN Framework

**Figure 4:**
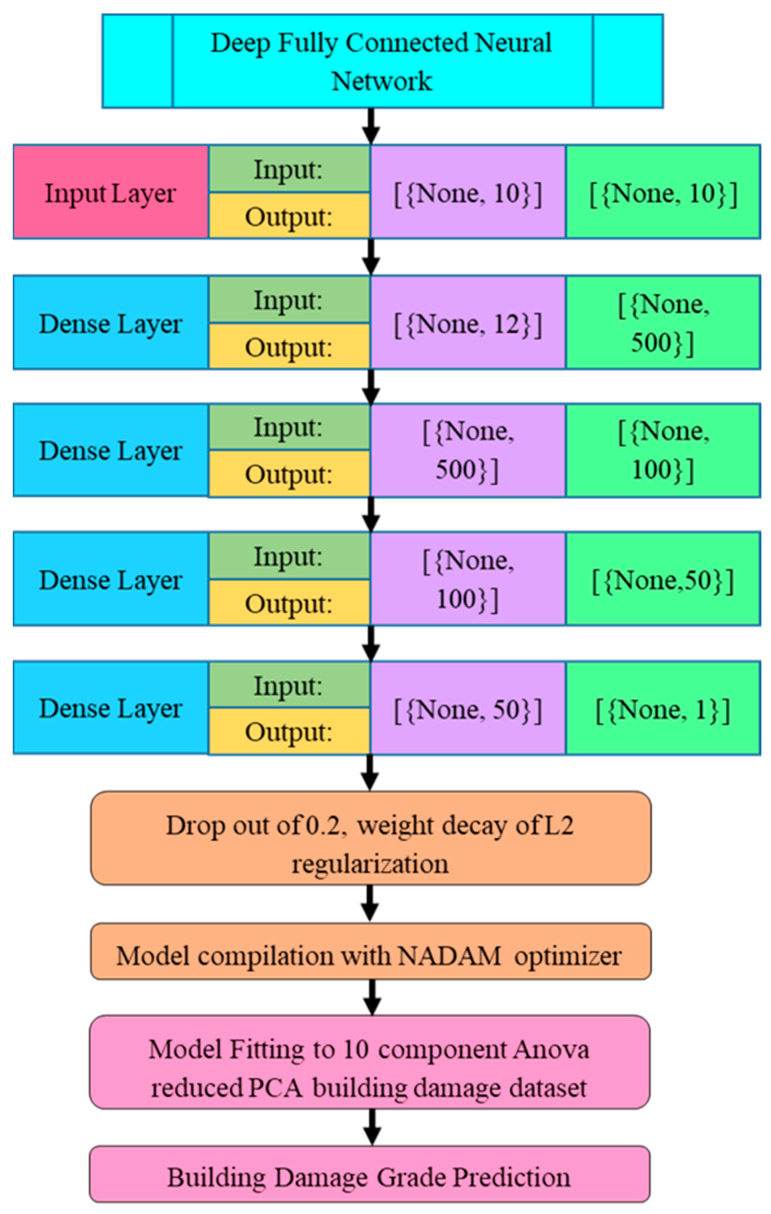
ASR-DFCNN model design and compilation workflow.

The layered framework of the fully connected ASR-DFCNN and model compilation flow is illustrated in Figure 4. The proposed ASR-DFCNN model was designed with a sequential keras model consisting of four dense layers, where the first three dense layers were fitted with the ReLU activation function, and the final dense layer was fitted with the tanh activation function with a dropout of 0.2. The ASR-DFCNN model was compiled with the NADAM optimizer with a weight decay of L2 regularization. The ASR-DFCNN model was trained with a learning rate of L2 regularized gradient descent function.

**Figure 5:**
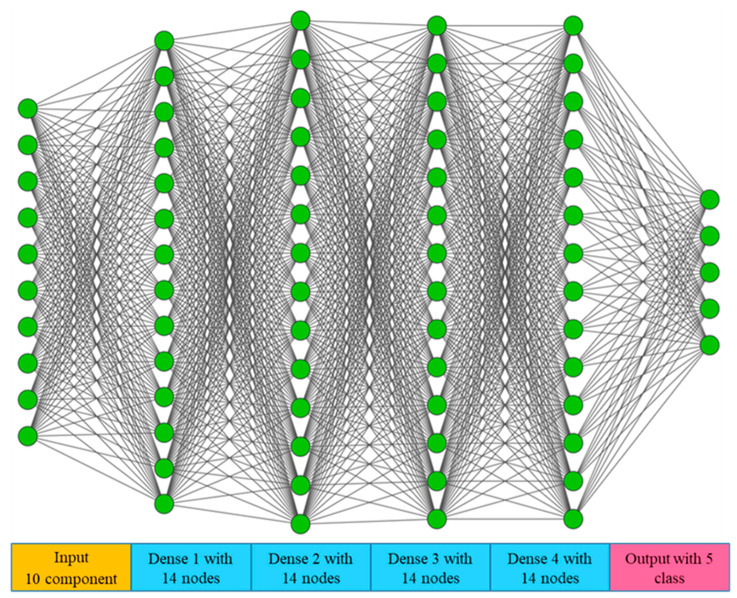
ASR-DFCNN architectural input and dense layer framework.

The ASR-DFCNN module network architecture, with input and dense layer framework, is shown in Figure 5, where the green circles represent each layer’s components. The model was fed with 10 input components at the input layer; convolution was conducted at four dense layers with 14 nodes and a single output layer that categorized five classes of damage grade. The performance of the proposed model was analyzed, and accuracy was compared with other classifier models.

After modeling the proposed framework, data were preprocessed as follows. The earthquake damage dataset with 25 independent features and one dependent feature “damage grade,” is represented in Equation (1).

(1) E={[e1,e2,e3,……….., e25], [D]},

The E represents the dataset, D the target variable, and [e1,e2,e3,……….., e25] the 25 independent variables. The feature encoding of categorical attributes in the dataset is shown in Equation (2), where ei is the encoded variable and U denotes the integer values for each category.

(2) ei=U→{0,1,2,…25}

After data encoding, the missing values in the dataset were filled with the mean values of the feature column using Equation (3):

(3) $$eR_{ij}=\frac{1}{n}\;\overset{m}{\underset{d=1}{\sum }}{\left(eR_{ij}\right)}^{d}$$

where “eR” is the estimated mean of each feature column in the dataset and ‘d’ is the total number of features designated as ‘25’. The resultant imputed data were subjected to feature scaling to maintain uniformity in values using Equation (4):

(4) $$E^{\prime }=\frac{1}{E}\overset{25}{\underset{e=1}{\sum }}{E^{\prime }}_{e}$$

where $E^{\prime }$ is the processed dataset over attributes e = 1, 2, …, 25, the independent features of the original dataset. The variance between the original and the imputed feature is estimated using Equation (5):

(5) $$EW^{\prime }=\frac{1}{E}\overset{26}{\underset{e=1}{\sum }}{E^{\prime }}_{e}=\frac{1}{E}\overset{26}{\underset{e=1}{\sum }}var({E^{\prime }}_{e})$$

where $EW^{\prime }\;$is the variance computed on the completed data. Impute dataset was generated by applying calculated feature variance from Equation (5) to each column of the dataset as shown in Equation (6).

(6) $$Impute=EW^{\prime }-\frac{1}{E-1}\overset{26}{\underset{e=1}{\sum }}({E^{\prime }}_{e}-var({E^{\prime }}_{e}))$$

The final preprocessed data ‘FinalData’ with no missing values and having the total variance generated using Equation (7).

(7) $$FinalData=EW^{\prime }+\left(\frac{e+1}{e}\right)\times Impute$$

The min–max normalization method was applied to normalize the encoded imputed final data using the formula as shown in Equation (8). $e_{i}{}^{\prime }$ is the normalized feature value that lies within the range of 0 and 1.

(8) $$e_{i}{}^{\prime }=\frac{0+\left(e_{i}-\min\left(e_{i}\right)\right)\left(1-0\right)}{\max\left(e_{i}\right)-\min\left(e_{i}\right)}$$

The preprocessed dataset was fitted to all classifier models before and after component scaling. The performance was assessed by cross-sectioning the training and testing data in 80:20, 70:30, and 60:40 ratios. Let variable eR denote the number of rows in the dataset, ‘d’ is the number of features, and variable ij represents the instances of the rows and columns. The principal component analysis was conducted by finding the covariance of two feature variables $COV\left(eR_{ij},eR_{pq}\right)$ in the dataset, as shown in Equation (9).

(9) $$COV\left(eR_{ij},eR_{pq}\right)=\frac{1}{e-1}\;\overset{25}{\underset{d=1}{\sum }}\left[\left(eR_{ij}{}_{d}\right)-{\left(eR_{ij}{}_{d}\right)}^{\prime }\right]\;\left[\left(eR_{pq}{}_{d}\right)-{\left(eR_{pq}{}_{d}\right)}^{\prime }\right]$$

The ANOVA test was conducted to find the essential features in the dataset for determining the building damage grade using Equations (10)–(12).

(10) $$X_{Anova}=\frac{Mean\;sum\;of\;squares\;of\;treatment\;\left[MST\right]}{Mean\;sum\;of\;squares\;of\;error\;\left[MSE\right]}$$

(11) $$MST=\overset{d}{\underset{e=1}{\sum }}\frac{\left(\frac{eR_{ij}{}^{2}}{d_{i}}\right)-{\left(\frac{eR_{pq}{}_{d}}{d_{i}}\right)}^{\prime }}{d=1}$$

(12) $$MSE=\overset{k}{\underset{i=1}{\sum }}\overset{d}{\underset{j=1}{\sum }}\left[eR_{ij}{}^{2}\right]-\overset{k}{\underset{i=1}{\sum }}\overset{d}{\underset{j=1}{\sum }}\left[\frac{eR_{ij}{}^{2}}{d_{i}}\right]$$

The proposed ASRDFCNN containing one input ‘InLayer’ with 10 components, four dense layers ‘DenseLayer’ with 14 nodes, and an output layer ‘OutputLayer’ with five classes are represented in Equations (13)–(16).

(13) ASRDFCNN=InLayer+4 Dense+OutLayer

(14) $$InLayer_{j}^{\left[i\right]}=\overset{10}{\underset{i=1}{\sum }}Input_{j,i}^{i}$$

(15) $$DenseLayer_{j}^{\left[i\right]}=\overset{14}{\underset{i=1}{\sum }}Dense_{j,i\;}^{i}Dense_{i}^{i+1}$$

(16) $$OutputLayer_{j}^{\left[i\right]}=\overset{1}{\underset{i=1}{\sum }}Output_{j,i}^{i}$$

The ASR-DFCNN was implemented using a sequential keras model with four dense layers, in which the first three dense layers were fitted with the ReLU activation function, and the final dense layer was fitted with the tanh activation function with a dropout of 0.2. The ASR-DFCNN model is compiled with a NADAM optimizer with the weight decay of L2 regularization. The ASR-DFCNN model is trained with a learning rate using the L2-regularized gradient descent function. The first three dense layers were activated with the ReLU activation function, and the weight initialization ‘W(t)’ of those first three dense layers having t as weight is shown in Equation (17).

(17) $$W\left(t\right)=\left\{\begin{array}{c} 0\;\;\;\;\;\;\;t\leq 0\\ t\;\;\;\;\;\;\;\;t>0 \end{array}\right\}$$

The final dense layer is fitted with the tanh activation function and the weight initialization W(t) of the final dense layer is given by Equation (18).

(18) $$W\left(t\right)=\frac{2}{1+e^{-2t}}-1$$

After designing the dense layer, the ASR-DFCNN model was compiled with weight decay $L2RGD_{i}\;$using the L2-regularized gradient descent function RG as shown through Equations (19)–(21).

(19) $$L2RGD_{i+1}=L2RGD_{i}+\eta \;\frac{\delta W\left(t\right)}{\delta L2RGD}-\frac{\eta \;\lambda }{\eta }RG$$

(20) $$RG=RG_{0}+\frac{\lambda }{2\eta }\underset{rg}{\sum }RG^{2}$$

(21) $$\lambda =\frac{\;\lambda^{\prime }}{c}RG$$

The ASR-DFCNN model was optimized using a NADAM optimizer as shown in Equations (22)–(25):

(22) $$GD_{t}=\nabla_{L2RGD_{t-1}}F\left(L2RGD_{t}-1\right)$$

(23) $$MGT_{t}=\mu \;MGT_{t-1}+\left(1-\mu \right)GD_{t}$$

(24) $$BCN_{t}=MV\;BCN_{t-1}+\left(1-\mathrm{MV}\right)GD_{t}{}^{2}$$

(25) $$LeR_{i}=LeR_{i-1}-\eta \;\frac{MGT_{t}}{\sqrt{BCN_{t}+\epsilon \;}}$$

where $GD_{t}$ is the gradient descent function at step ‘t’, $MGT_{t}$ is the sum of the previous gradient vectors, μ is the decay constant, $BCN_{t}$ is the initialization bias correction term, MV is the momentum vector, $LeR_{i}$ is the learning rate, η is the learning rate decay, and λ is the loss error. The model performance was measured using accuracy which denotes the ratio of number of correct predictions to the total number of predictions, as given in Equation (26).

(26) $$Accuracy=\frac{True\;Positives+True\;Negatives}{True\;Positives+True\;Negatives+False\;Positives+False\;Negatives}$$

## 4. Implementation

The building earthquake damage dataset used for implementation was taken from the KAGGLE repository. Twenty-six attributes collected from 762,106 buildings were utilized for building damage grade prediction. The attributes describe the structure of the building, the nature of material used, and land and surface conditions. Among the 26 listed features, damage_grade was the target variable, and the remaining 25 features were considered independent variables. The details of the data are tabulated in Table 1. Our target variable had five labels of damage grade, namely, ‘Minor’, ‘Moderate’, ‘Heavy’, ‘Extremely severe’, and ‘Disaster’. The dataset was subjected to data preprocessing, such as filling in the missing values, categorical encoding, feature scaling, and normalization. Python programming was used for the model implementation with pandas and keras libraries.

### Prescriptive Data Analysis of Building Damage

**Figure 6:**
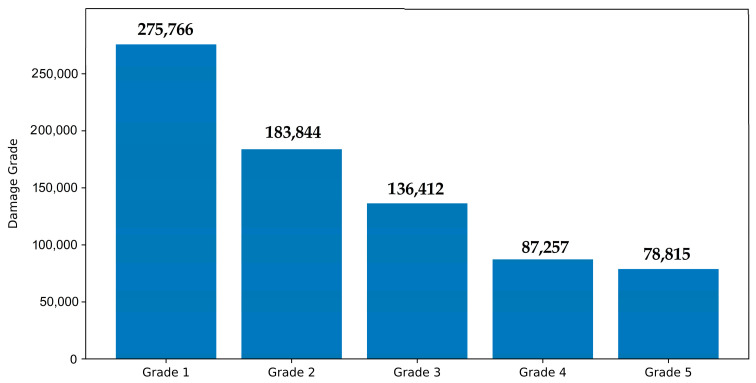
Damage grade exploration in the dataset.

**Figure 7:**
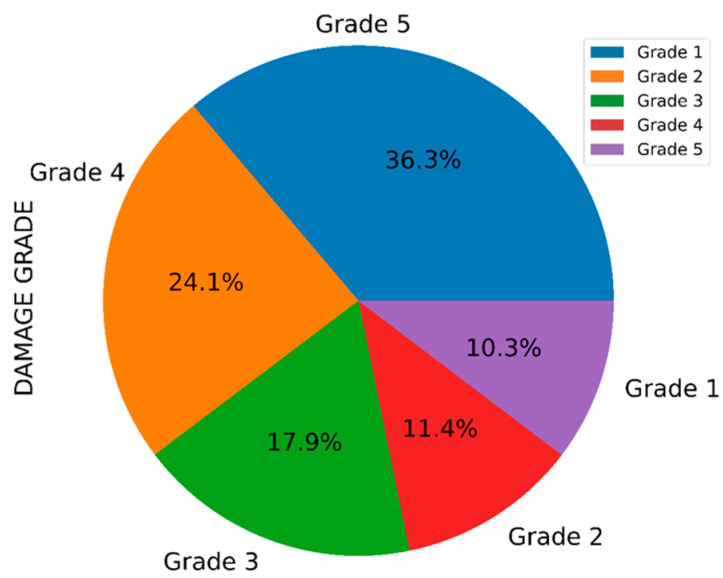
Damage grade distribution in the dataset.

The exploratory data analysis of the target variable ‘damage_grade’ is shown in Figure 6 and Figure 7. From the distribution, it was observed that each grade occurs more frequently. Grade 5 appears more extensively in the samples, and Grade 1 occurs the least often. Evidently, this classification problem with various grades accounts for drastically different observation proportions. The distribution of damage grade was completed based on the following description.

Grade 1: Minor damage (a few walls exhibiting hairline fractures).

Grade 2: Moderate damage (big plaster parts collapsing).

Grade 3: Heavy damage (large, prolonged wall fractures).

Grade 4: Extremely severe damage (serious wall collapse).

Grade 5: Disaster (total collapse).

**Figure 8:**
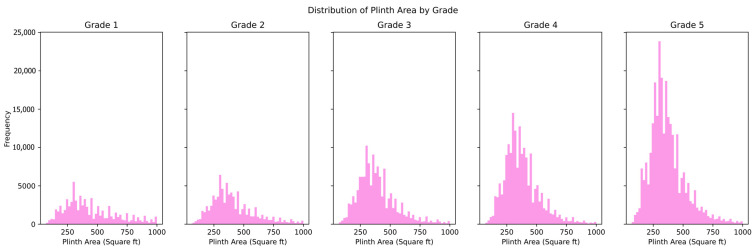
Distribution of plinth area to damage grade in the dataset.

**Figure 9:**
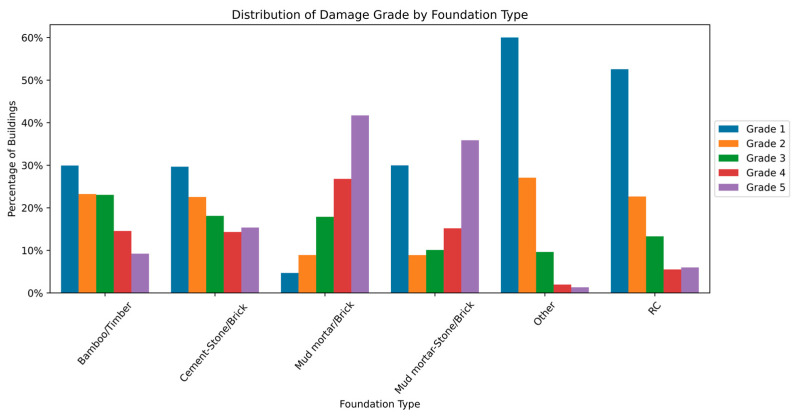
Dissemination of damage grade to foundation type.

**Figure 10:**
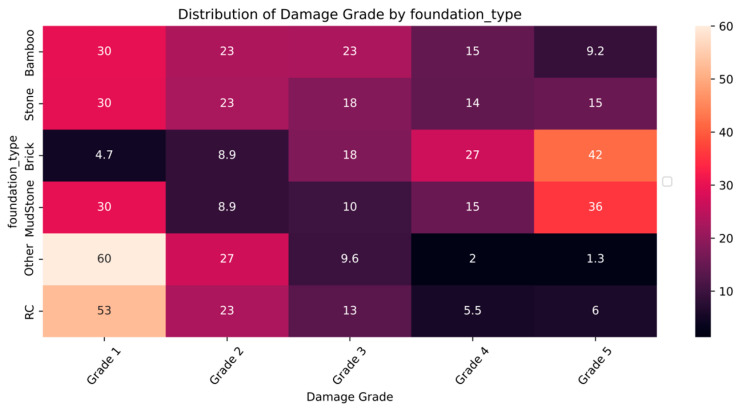
Statistics of damage grade to foundation type.

**Figure 11:**
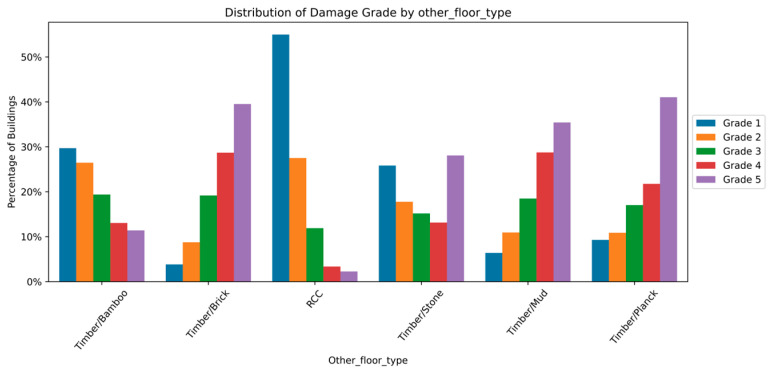
Dissemination of damage grade to other floor type.

**Figure 12:**
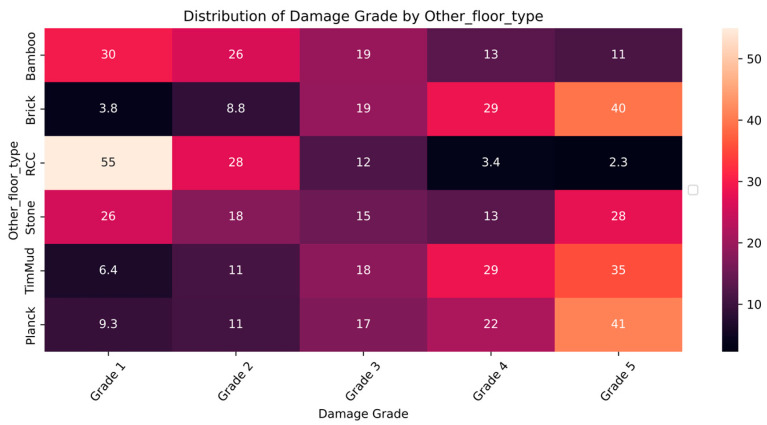
Statistics of damage grade to other floor type.

**Figure 13:**
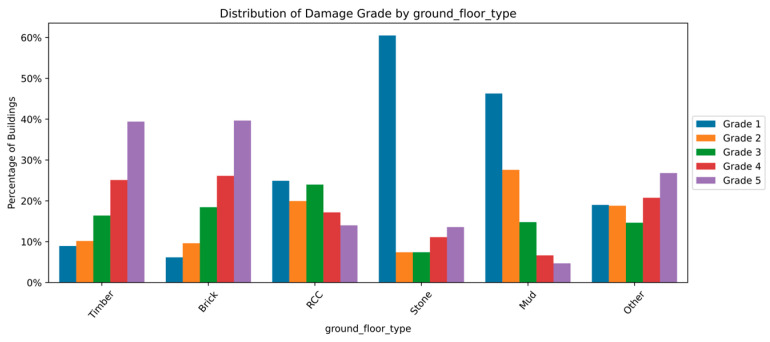
Dissemination of damage grade to ground floor type.

**Figure 14:**
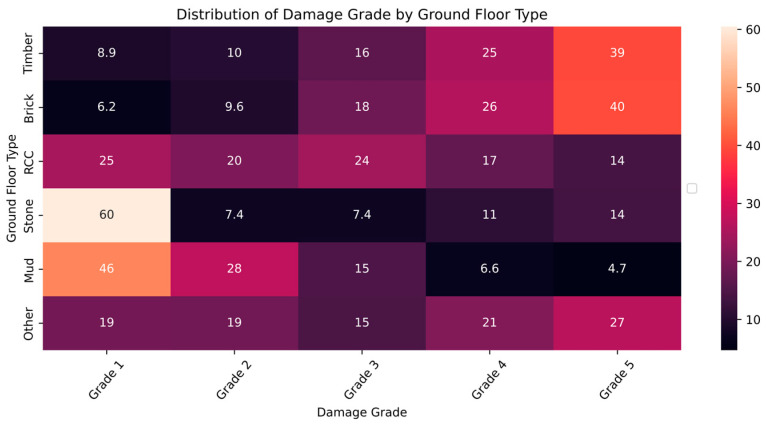
Statistics of damage grade to ground floor type.

**Figure 15:**
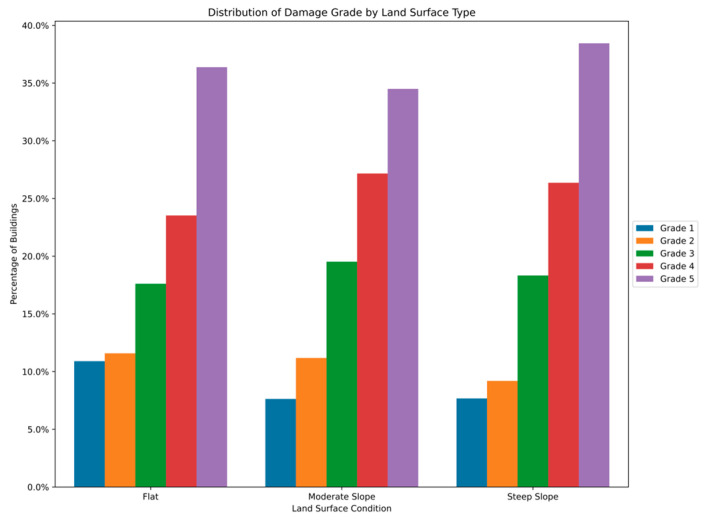
Dissemination of damage grade to land surface.

**Figure 16:**
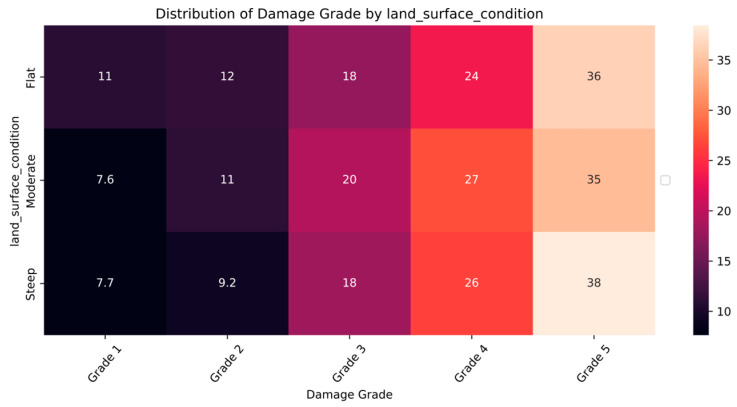
Statistics of damage grade to land surface.

**Figure 17:**
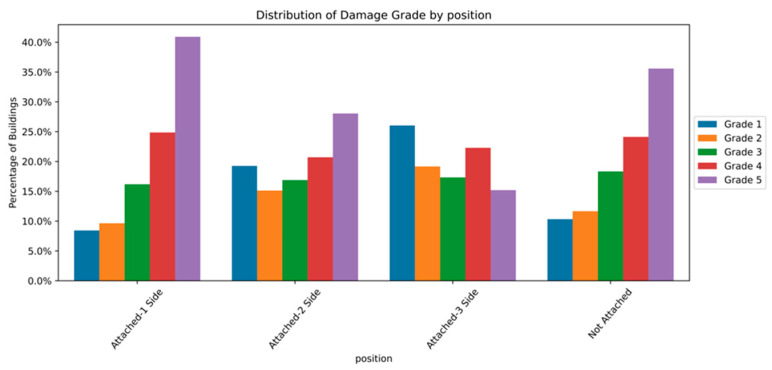
Dissemination of damage grade by position.

**Figure 18:**
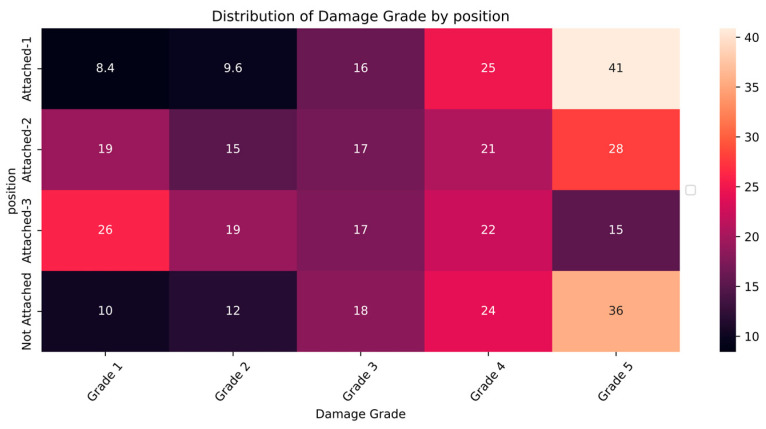
Statistics of damage grade by position.

**Figure 19:**
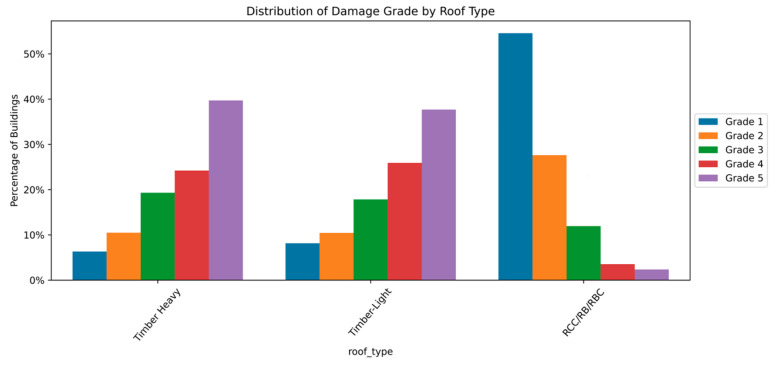
Dissemination of damage grade by roof type.

**Figure 20:**
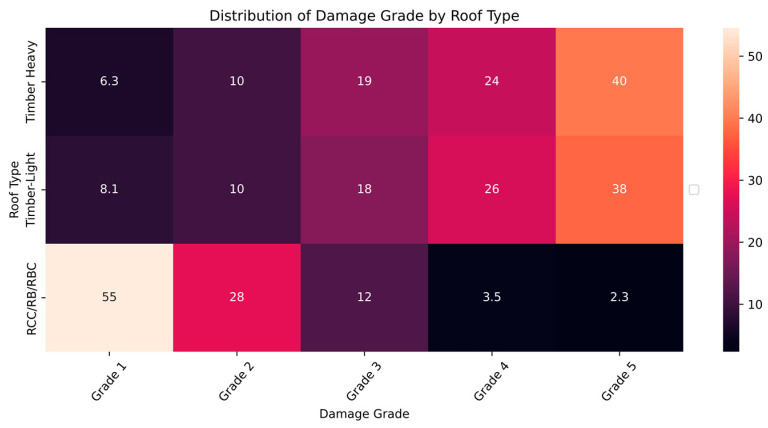
Statistics of damage grade by roof type.

The distribution and the statistics damage grade to plinth area, foundation type, other floor types, ground floor types, land surface type, position, and roof types are shown below in Figure 8, Figure 9, Figure 10, Figure 11, Figure 12, Figure 13, Figure 14, Figure 15, Figure 16, Figure 17, Figure 18, Figure 19 and Figure 20. The distribution of damage grade to the plinth area, depicted in Figure 8, conveys that many buildings with higher plinth areas fall under the category of ‘Grade 5’ compared to the other damage grades.

The statistical distribution of damage grade to foundation type is shown in Figure 9 and Figure 10. The foundation_type is a categorical variable with six materials used in building foundations. It was observed that ‘Grade 5’ damages were less likely across all foundations. ‘Grade 1’ damage occurred in almost all foundation types. The buildings made of mud and brick showed disastrous effects, whereas bamboo and cement types exhibited an inverse relation to damage grade. The RC and other floor types showed similar patterns of damage, which might be due to a smaller sample size. Figure 10 shows the distribution of damage grades across the foundation types in percentage.

The statistical distribution of damage grade to floor types other than the ground floor is depicted in Figure 11 and Figure 12. The data illustrated that RCC, brick, and plank were widely used as flooring materials. Also, the graph demonstrated that the catastrophic damage was minor in RCC floors, whereas it was high in timber-based materials. Almost-damaged Grades 2, 3, and 4 were likely to appear on all types of floors. The floor type feature revealed a strong association with damage grades and thus was considered for classification.

Figure 13 and Figure 14 illustrate the statistical data distribution of damage grade to ground floor type. Mud was most used for ground floors, followed by brick and RCC. The damage grade distribution across the ground floor differed from other floor damages. Devasting damages were prominent among buildings with timber and brick, whereas stone floors were prone to Grade 1 damages. Depending on the materials used to make the ground, the relationship between ground floor type and damage grade varies significantly. The observations imply that ground_floor_type has significant forecasting potential.

The data distribution of building damages to the nature of the land surface is shown in Figure 15 and Figure 16. The instances show that moderate and steep slopes had significant damage, and flat surfaces had lesser damage grades. The data distribution pattern is strongly related to the land surface and damage grade features.

The position attribute denotes the attachment of other buildings at their sides. The building may be attached to between one and three sides of its location. Most buildings in the dataset were represented by the “Not attached” position. Figure 17 and Figure 18 show that the buildings with an attachment on one side and with no attachment show a similar relationship between position and damage grade. The damages are severe in the one-side and no-attachment types of buildings. The patterns of the buildings with two- and three-sided attachments were less vulnerable to devasting damages.

The data distribution among roof type and damage grade summarizes that building roofs made of timber are prone to damage of all grades in increasing order from simple to disaster. The building roofs made of RCC were less affected by the earthquake. The RCC roof might be considered an alternative roofing material to prevent heavy damage during earthquakes. The data distribution of roof material and damage grade is illustrated in Figure 19 and Figure 20.

**Figure 21:**
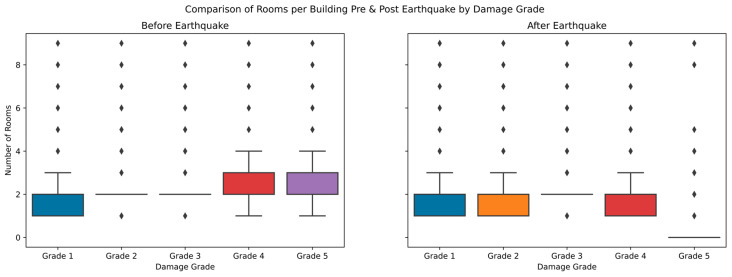
Comparison of number of rooms before and after earthquake to damage grade.

The impact analysis of the damage to the number of rooms in the building before and after the earthquake is illustrated in Figure 21. The data distribution indicates that damage grades were observed in an average of one to four rooms. There were some outliers where damage was caused in all building rooms, indicating that the damages depended on the center of seismic activity. The number of rooms is unimportant in grading the earthquake damage categorization.

**Figure 22:**
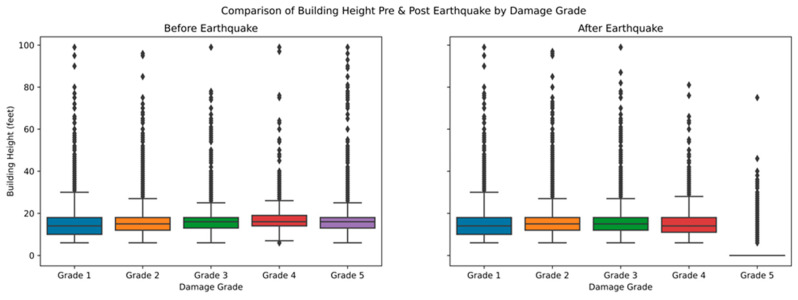
Building height comparisons before and after earthquake to damage grade.

The distribution of building heights to damage grade before and after the earthquake was analyzed with the graph shown in Figure 22. The general distribution of building heights for the damage Grades 1–3 remained the same before and after the earthquake. Damage Grade 5 dropped to zero for most of the buildings, implying that these buildings completely collapsed, whereas damage Grade 4 showed a modest height loss after an earthquake.

The prescriptive data exploratory analysis of the features is shown in Table 2. From the prescriptive analysis, the metrics like mean, standard deviation, and minimum and maximum values of the dataset features are extracted. The statistics show that the plinth area projects the highest mean, standard deviation, and minimum and maximum values compared to the rest of the features.

## 5. Results and Discussion

**Figure 23:**
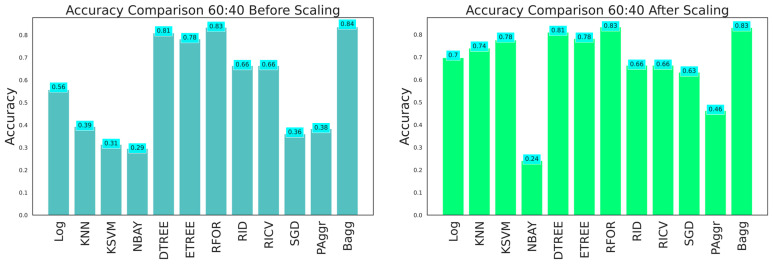
Accuracy comparison for 60:40 training and testing data.

**Figure 24:**
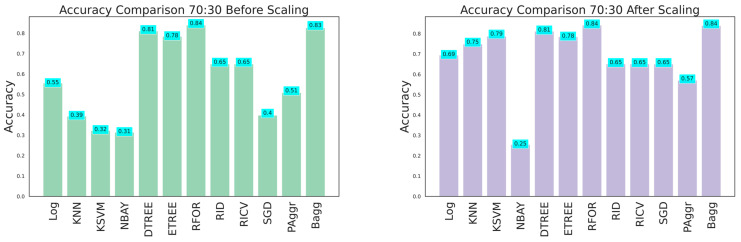
Accuracy comparison for 70:30 training and testing data.

**Figure 25:**
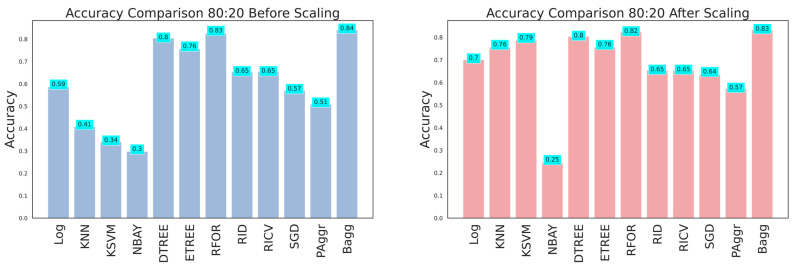
Accuracy comparison for 80:20 training and testing data.

The dataset with 26 features was fitted to all the classifiers to grade the damage before and after feature scaling. The ML classifier models such as logistic regression (LReg), K nearest neighbors (KNN), kernel support vector machine (KSVM), Gaussian naive Bayes (GNB), decision tree (Dtree), extra tree (Etree), random forest (RFor), ridge classifier (Ridge), RidgeClassifierCV (RCV), stochastic gradient descent (SGD), passive aggressive (PAg), and bagging (Bagg) were implemented. The exploratory data analysis aided in understanding the pattern of data distribution. Further, an adequate dataset was needed to construct an efficient damage classification model. Thus, the performance of the classifiers was analyzed at the train–test split with various ratios (60:40, 70:30, and 80:20), with and without feature scaling, and feature selection using the ANOVA test and PCA. Initially, the performance analysis of the models was conducted on the original data considering the training and testing dataset in the 60:40, 70:30, and 80:20 ratios. The evaluation metrics obtained are tabulated in Table 3, Table 4 and Table 5, and the graphical plots are shown in Figure 23, Figure 24 and Figure 25. The results proved that the model’s classification accuracy and precision significantly improved after implementing feature scaling. The performance indicated that feature scaling eliminates feature biasing and enables the machine learning models to interpret the features on a similar scale. A comparison of the performance of models with various train–test split ratios showed that the model performed better when the dataset was divided into an 80:20 ratio. The accuracy of the models improved when the training data were increased.

**Figure 26:**
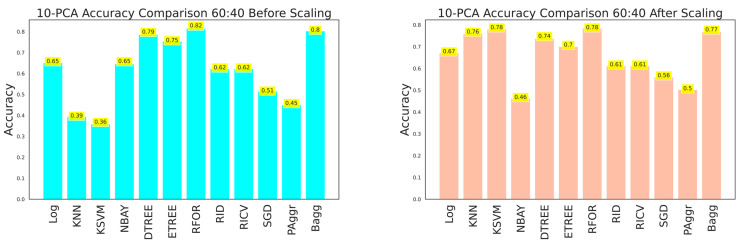
Accuracy comparison of 10-component PCA dataset for 60:40 training and testing data.

**Figure 27:**
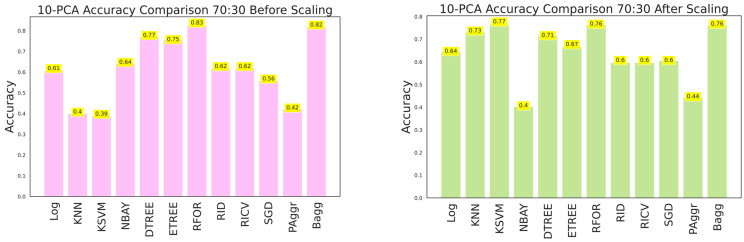
Accuracy comparison of 10-component PCA dataset for 70:30 training and testing data.

**Figure 28:**
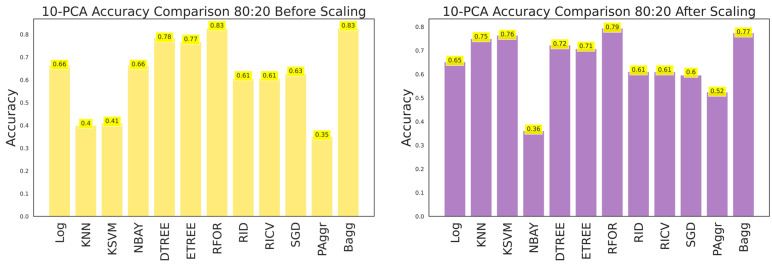
Accuracy comparison of 10-component PCA dataset for 80:20 training and testing data.

The effectiveness of the dataset was improved by selecting the most prominent features that contributed to categorizing the building damage. A 10-component PCA was applied to select the essential features from the raw dataset before and after feature scaling. The resultant dataset was fitted to the classifiers, and performances were compared. Evaluation metrics were obtained using cross-sectioning training and testing data with 80:20, 70:30, and 60:40 ratios, as shown in Table 6, Table 7 and Table 8 and Figure 26, Figure 27 and Figure 28, respectively. The results verified that model performance was further improved when fitted with significant features than on the entire dataset.

### 5.1. ANOVA Test Analysis

The ANOVA test was used to determine the influence of the independent features on the target variable. The ANOVA test analyzes the dataset’s features by comparing the null and alternate hypotheses. The PR value implies the probability of obtaining the observed value believing that the null hypothesis is true. F denotes the ratio between the variability among the variables and within group variables. An F value less than 0.05 indicates that the variable highly influenced the target variable. The degree of freedom (df) represents the number of independent variables considered to check the variability among the group. The preprocessed dataset with 25 input variables was subjected to an ANOVA test, and the results are tabulated in Table 9. The features plinth_area_sq_ft, position, has_superstructure_cement_mortar_stone, has_superstructure_1_mortar_brick, has_super_4_non_engineered, has_superstructure_ stone_flag, and has_superstructure_4 _ engineered have a PR(>F) >0.05, and determined insignificance. Thus, the dataset was refined by eliminating the irrelevant features to form an ANOVA-reduced building damage dataset.

### 5.2. PCA ANOVA-Reduced Predictive Analysis

**Figure 29:**
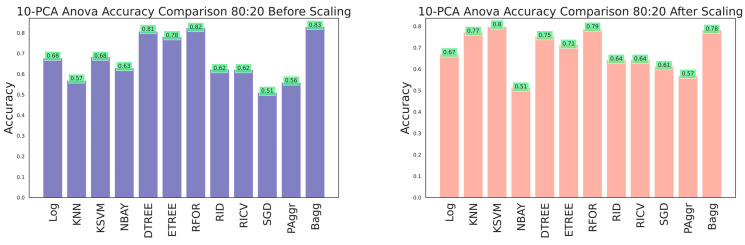
Accuracy of 10-component PCA ANOVA dataset for 80:20 training and testing data.

**Figure 30:**
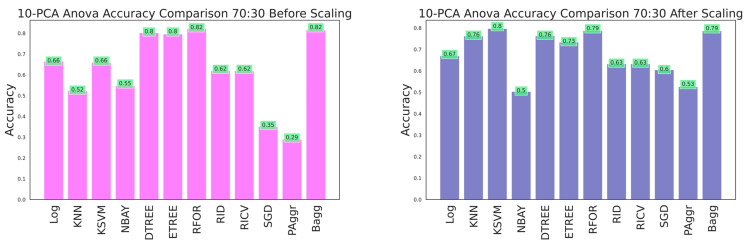
Accuracy of 10-component PCA ANOVA dataset for 70:30 training and testing data.

**Figure 31:**
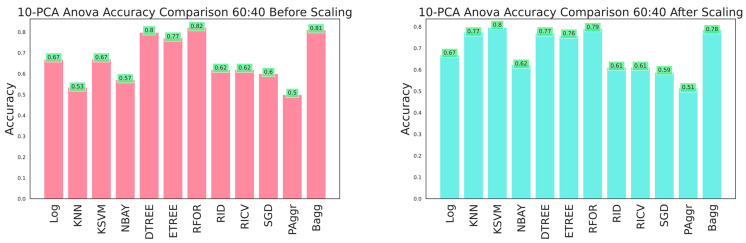
Accuracy of 10-component PCA ANOVA dataset for 60:40 training and testing data.

The ANOVA-reduced building damage dataset contains 18 input features. The effectiveness of the dataset was further improved by applying a 10-component PCA, which filtered the top 10 significant features that intend to predict the building damage grade. The 10-component PCA ANOVA-reduced dataset was fitted to all the classifiers, and performances were recorded. The performance of the classifiers on feature scaling and with varied split ratios were listed in Table 10, Table 11 and Table 12 and shown in Figure 29, Figure 30 and Figure 31.

The extensive experimentation provided the following perceptions: 1. The ML model could perform a task more efficiently when the data used for training was considerable. The 80:20 split ratio fit well while dividing the dataset for training and testing. 2. The model interpretation of the input features would be unbiased if a similar scaling was adopted across all the features. 3. The model’s performance can be enriched if trained with relevant and significant features that determine the output. Thus, a 10-component PCA ANOVA-reduced dataset was created to build an earthquake damage prediction model.

### 5.3. Proposed ANOVA-Statistic-Reduced Deep Fully Connected Neural Network

**Figure 32:**
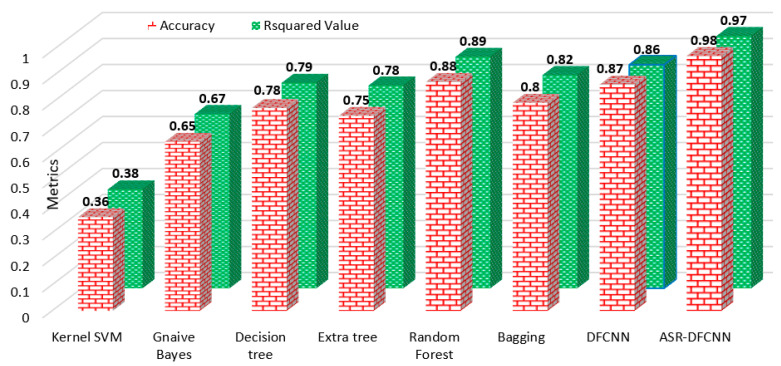
Accuracy and R-squared value of ASR-DFCNN and other classifier models.

The deep, fully connected neural network model that accepts 10 input components and outputs any of the five classes of damage grade was implemented using Python programming on an NVIDIA Tesla V100 GPU workstation. The proposed ASR-DFCNN was trained and tested with 80% and 20% of the data. The model was executed with a batch size of 64 for 30 epochs. The proposed model was validated by comparing the accuracy and R-squared values with all other classifiers on the reduced dataset. Also, the designed DFCNN model was implemented with the raw dataset. The observations in Table 13 and Figure 32 proved that the proposed ASR-DFCNN model attained a greater accuracy of 98% and R-squared 97% than other models. The DFCNN model, when trained with raw data, attained only 87% accuracy, thus proving the importance of feature selection for building an efficient model.

## 6. Conclusions

The presented research attempted to grade the type of building damage caused by an earthquake by analyzing the essential features. The main objective of this research is to investigate how well the performance of the deep, fully connected neural network can be improved by tuning the input features and parameters of model compilation. The contribution of the research was two-fold. The first was identifying essential data processing techniques on input features to build a competent dataset. The second focus was designing the ASR-DFCNN model-based architecture that efficiently classified the earthquake-affected building’s damage grade compared to existing ML classifiers. The challenges in building the proposed ASR-DFCNN were input feature selection and opting the best the activation and optimization functions to improve model accuracy. The earthquake damage dataset with 26 attributes describing 762,106 buildings was used to train the proposed ASR-DFCNN model, which fulfilled this research work’s requirements and outperformed the existing DFCNN and classifier models. Initially, the raw dataset without feature scaling and selection was directly fitted to the classifiers in data split ratios of 60:40, 70:30, and 80:20 for training and testing. The performance analysis concluded that the accuracy of the models without feature scaling lay between 50% to 70%. The performance increased with feature scaling and splitting data with an 80:20 ratio for training and testing. However, when the same classifiers were applied with a 10-component PCA-reduced dataset, the accuracy of the models showed better improvement. After the ANOVA test implementation, the features that produced the PR (>F) > 0.05 were considered insignificant and eliminated from the dataset. Thus, the size of the dataset was reduced from 25 to 18 input features. A 10-component PCA was applied to the ANOVA-reduced dataset to select the top 10 input features contributing significantly to damage prediction. The results exhibited that the bagging classifier with the reduced dataset produced the greatest accuracy of 83% among all the classifiers considering an 80:20 ratio of data for the training and testing phases. To enhance the performance of prediction, a deep fully connected convolutional neural network (DFCNN) was implemented with a reduced dataset (ASR).

The proposed ASR-DFCNN model was designed with the sequential keras model with four dense layers, with the first three dense layers fitted with the ReLU activation function and the final dense layer fitted with a tanh activation function with a dropout of 0.2. The ASR-DFCNN model was compiled with a NADAM optimizer with the weight decay of L2 regularization. The research model fitted with an appropriate activation function to the dense hidden layers and model optimizers reduced the loss and produced improved accuracy in damage grade classification. The ASR-DFCNN model was trained and tested with the resultant dataset and validated by comparing its performance with all other classifier models. The results proved that the ASR-DFCNN model outperformed other models by achieving 98% accuracy and 97% R-squared value. Despite the proposed ASR-DFCNN model’s remarkable performance, it is still challenging for researchers to fine-tune the sampling ratios of data features by experimenting with various oversampling or under-sampling methods. This research work could also be further enriched by extending the outlier analysis and extraction of the significant data features.

## Figures and Tables

**Table 1:** Building earthquake damage dataset information.

S.No	Column	Non-Null Values	Count	Dtype
1.	count_floors_pre_eq	762,106	non-null	int64
2.	count_floors_post_eq	762,106	non-null	int64
3.	age_building	762,106	non-null	int64
4.	plinth_area_sq_ft	762,106	non-null	int64
5.	height_ft_pre_eq	762,106	non-null	int64
6.	height_ft_post_eq	762,106	non-null	int64
7.	land_surface_condition	762,106	non-null	int64
8.	foundation_type	762,106	non-null	object
9.	roof_type	762,106	non-null	int64
10.	ground_floor_type	762,106	non-null	int64
11.	3_floor_type	762,106	non-null	int64
12.	position	762,105	non-null	float64
13.	plan_configuration	762,105	non-null	object
14.	has_superstructure_adobe_1	762,106	non-null	int64
15.	has_superstructure_1_mortar_stone	762,106	non-null	int64
16.	has_superstructure_stone_flag	762,106	non-null	int64
17.	has_superstructure_cement_mortar_stone	762,106	non-null	int64
18.	has_superstructure_1_mortar_brick	762,106	non-null	int64
19.	has_superstructure_cement_mortar_brick	762,106	non-null	int64
20.	has_superstructure_5	762,106	non-null	int64
21.	has_superstructure_bamboo	762,106	non-null	int64
22.	has_superstructure_4_non_engineered	762,106	non-null	int64
23.	has_superstructure_4_engineered	762,106	non-null	int64
24.	has_superstructure_3	762,106	non-null	int64
25.	condition_post_eq	762,106	non-null	object
26.	damage_grade	762,094	non-null	object

**Table 2:** Prescriptive data analysis of building earthquake damage dataset.

Features	Mean	STD	min	25%	50%	75%	max
count_floors_pre_eq	1.867975207	0.607971459	1	1	2	2	9
count_floors_post_eq	1.859090909	0.608865412	1	1	2	2	9
age_building	19.41508264	21.4985166	0	8	15	25	999
plinth_area_sq_ft	333.3260331	116.4665702	70	260	322	391	2850
height_ft_pre_eq	15.07768595	4.37791122	6	12	15	18	35
height_ft_post_eq	15.03677686	4.439751992	6	12	15	18	48
land_surface_condition	0.216528926	0.562497767	0	0	0	0	2
foundation_type	1.956818182	0.289648867	0	2	2	2	4
roof_type	0.870247934	0.345165801	0	1	1	1	2
ground_floor_type	0.951652893	0.430469684	0	1	1	1	5
floor_type	2.181404959	1.464939601	0	1	1	3	5
position	2.879545455	0.584425365	0	3	3	3	3
plan_configuration	6.850826446	0.889457053	1	7	7	7	10
has_superstructure_adobe_1	0.126033058	0.331920908	0	0	0	0	1
has_superstructure_1_mortar_stone	0.955578512	0.206050943	0	1	1	1	1
has_superstructure_stone_flag	0.004132231	0.06415611	0	0	0	0	1
has_super_cement_mortar_stone	0.002479339	0.049736333	0	0	0	0	1
has_superstructure_1_mortar_brick	0.000619835	0.024891334	0	0	0	0	1
has_supers_cement_mortar_brick	0.002479339	0.049736333	0	0	0	0	1
has_superstructure_5	0.060123967	0.237740938	0	0	0	0	1
has_superstructure_bamboo	0.040289256	0.196657119	0	0	0	0	1
has_super_4_non_engineered	0.002272727	0.047623845	0	0	0	0	1
has_superstructure_4_engineered	0	0	0	0	0	0	0
has_superstructure_3	0.004545455	0.067273535	0	0	0	0	1
condition_post_eq	4.169834711	2.270432966	2	2	3	7	8
damage_grade	1.488016529	1.1720469	0	0	1	3	3
technical_solution_proposed	1.536570248	1.122348734	0	1	1	3	3

**Table 3:** Damage grade classifier performance indices 60:40 before and after feature scaling.

Classifier	Before Feature Scaling					After Feature Scaling				
	Precision	Recall	F-Score	Accuracy	Runtime	Precision	Recall	F-Score	Accuracy	Runtime
LReg	0.57	0.58	0.55	0.56	0.21	0.71	0.70	0.69	0.70	0.19
KNN	0.42	0.41	0.40	0.39	0.07	0.77	0.76	0.76	0.74	0.18
KSVM	0.31	0.34	0.28	0.31	1.26	0.83	0.79	0.78	0.78	0.60
GNB	0.58	0.30	0.29	0.29	0.00	0.58	0.25	0.23	0.24	0.00
Dtree	0.80	0.80	0.80	0.81	0.02	0.80	0.80	0.80	0.81	0.01
Etree	0.76	0.76	0.76	0.78	0.01	0.76	0.76	0.76	0.78	0.00
RFor	0.83	0.83	0.83	0.83	0.10	0.83	0.82	0.83	0.83	0.10
Ridge	0.68	0.65	0.63	0.66	0.08	0.68	0.65	0.63	0.66	0.00
RCV	0.68	0.65	0.63	0.66	0.04	0.68	0.65	0.63	0.66	0.02
SGD	0.78	0.43	0.36	0.49	0.13	0.67	0.67	0.66	0.63	0.10
PAg	0.42	0.45	0.37	0.55	0.07	0.55	0.51	0.52	0.58	0.03
Bagg	0.85	0.85	0.85	0.83	0.11	0.85	0.85	0.85	0.83	0.13

**Table 4:** Damage grade classifier performance indices 70:30 before and after feature scaling.

Classifier	Before Feature Scaling					After Feature Scaling				
	Precision	Recall	F-Score	Accuracy	Runtime	Precision	Recall	F-Score	Accuracy	Runtime
Lreg	0.63	0.55	0.52	0.55	0.18	0.71	0.70	0.69	0.69	0.19
KNN	0.42	0.40	0.39	0.39	0.08	0.77	0.76	0.76	0.75	0.16
KSVM	0.30	0.32	0.26	0.32	1.02	0.83	0.79	0.78	0.79	0.58
GNB	0.60	0.31	0.30	0.31	0.01	0.58	0.25	0.23	0.25	0.00
Dtree	0.81	0.81	0.81	0.81	0.02	0.80	0.80	0.80	0.81	0.01
Etree	0.79	0.78	0.79	0.78	0.01	0.76	0.76	0.76	0.78	0.00
Rfor	0.84	0.84	0.84	0.84	0.09	0.83	0.82	0.83	0.84	0.10
Ridge	0.68	0.65	0.63	0.65	0.01	0.68	0.65	0.63	0.65	0.01
RCV	0.68	0.65	0.63	0.65	0.01	0.68	0.65	0.63	0.65	0.01
SGD	0.59	0.27	0.14	0.41	0.14	0.68	0.67	0.65	0.63	0.07
Pag	0.55	0.19	0.13	0.32	0.04	0.48	0.47	0.45	0.56	0.01
Bagg	0.83	0.83	0.83	0.83	0.09	0.84	0.83	0.83	0.83	0.12

**Table 5:** Damage grade classifier performance indices 80:20 before and after feature scaling.

Classifier	Before Feature Scaling					After Feature Scaling				
	Precision	Recall	F-Score	Accuracy	Runtime	Precision	Recall	F-Score	Accuracy	Runtime
LReg	0.52	0.56	0.53	0.56	0.18	0.70	0.70	0.69	0.70	0.14
KNN	0.41	0.39	0.39	0.39	0.10	0.75	0.74	0.74	0.74	0.26
KSVM	0.31	0.31	0.24	0.31	0.80	0.81	0.78	0.77	0.78	0.42
GNB	0.57	0.29	0.28	0.29	0.01	0.57	0.24	0.21	0.24	0.00
Dtree	0.81	0.81	0.81	0.81	0.01	0.81	0.81	0.81	0.81	0.02
Etree	0.78	0.78	0.78	0.78	0.01	0.78	0.78	0.78	0.78	0.02
RFor	0.82	0.82	0.83	0.82	0.08	0.82	0.82	0.83	0.82	0.07
Ridge	0.68	0.66	0.64	0.66	0.01	0.68	0.66	0.64	0.66	0.00
RCV	0.68	0.66	0.64	0.66	0.03	0.68	0.66	0.64	0.66	0.02
SGD	0.57	0.34	0.22	0.34	0.12	0.64	0.64	0.63	0.64	0.07
PAg	0.64	0.30	0.16	0.30	0.04	0.60	0.60	0.59	0.60	0.03
Bagg	0.84	0.84	0.84	0.84	0.08	0.83	0.83	0.83	0.83	0.10

**Table 6:** Damage grade PCA classifier performance indices 60:40 before and after feature scaling.

Classifier	Before Feature Scaling					After Feature Scaling				
	Precision	Recall	F-Score	Accuracy	Runtime	Precision	Recall	F-Score	Accuracy	Runtime
LReg	0.68	0.64	0.63	0.66	0.17	0.70	0.68	0.67	0.67	0.06
KNN	0.42	0.41	0.40	0.39	0.05	0.77	0.76	0.76	0.76	0.15
KSVM	0.37	0.41	0.37	0.36	0.85	0.84	0.80	0.80	0.78	0.43
GNB	0.61	0.55	0.55	0.65	0.00	0.55	0.50	0.51	0.46	0.00
Dtree	0.80	0.79	0.79	0.79	0.10	0.77	0.77	0.77	0.72	0.10
Etree	0.75	0.75	0.75	0.75	0.00	0.73	0.72	0.72	0.71	0.01
RFor	0.84	0.84	0.84	0.82	0.30	0.84	0.84	0.84	0.78	0.33
Ridge	0.66	0.63	0.61	0.62	0.00	0.68	0.65	0.62	0.61	0.01
RCV	0.66	0.63	0.61	0.62	0.02	0.68	0.65	0.62	0.61	0.01
SGD	0.67	0.63	0.62	0.60	0.12	0.64	0.64	0.62	0.62	0.08
PAg	0.55	0.55	0.55	0.39	0.01	0.51	0.46	0.45	0.55	0.02
Bagg	0.84	0.84	0.84	0.81	0.35	0.79	0.79	0.79	0.76	0.36

**Table 7:** Damage grade PCA classifier performance indices 70:30 before and after feature scaling.

Classifier	Before Feature Scaling					After Feature Scaling				
	Precision	Recall	F-Score	Accuracy	Runtime	Precision	Recall	F-Score	Accuracy	Runtime
LReg	0.66	0.64	0.63	0.60	0.12	0.68	0.66	0.65	0.64	0.10
KNN	0.41	0.40	0.39	0.40	0.08	0.75	0.74	0.74	0.73	0.13
KSVM	0.35	0.39	0.34	0.39	0.71	0.83	0.79	0.79	0.76	0.36
GNB	0.60	0.54	0.54	0.64	0.00	0.57	0.55	0.54	0.40	0.00
Dtree	0.80	0.79	0.79	0.77	0.09	0.76	0.76	0.76	0.72	0.09
Etree	0.72	0.72	0.72	0.75	0.01	0.70	0.69	0.69	0.68	0.01
RFor	0.84	0.84	0.84	0.83	0.30	0.84	0.84	0.84	0.77	0.33
Ridge	0.66	0.63	0.60	0.62	0.01	0.70	0.66	0.63	0.59	0.01
RCV	0.66	0.63	0.60	0.62	0.01	0.70	0.66	0.63	0.59	0.00
SGD	0.56	0.54	0.54	0.49	0.07	0.63	0.62	0.60	0.58	0.06
PAg	0.51	0.47	0.48	0.44	0.02	0.42	0.40	0.39	0.56	0.01
Bagg	0.83	0.83	0.83	0.81	0.31	0.78	0.77	0.77	0.75	0.32

**Table 8:** Damage grade PCA classifier performance indices 80:20 before and after feature scaling.

Classifier	Before Feature Scaling					After Feature Scaling				
	Precision	Recall	F-Score	Accuracy	Runtime	Precision	Recall	F-Score	Accuracy	Runtime
LReg	0.65	0.64	0.63	0.65	0.14	0.69	0.67	0.66	0.65	0.04
KNN	0.41	0.39	0.39	0.40	0.08	0.76	0.76	0.76	0.74	0.14
KSVM	0.33	0.36	0.31	0.41	0.47	0.80	0.78	0.77	0.76	0.25
GNB	0.65	0.65	0.64	0.66	0.00	0.52	0.46	0.44	0.35	0.00
Dtree	0.79	0.79	0.79	0.78	0.06	0.73	0.73	0.73	0.73	0.05
Etree	0.75	0.75	0.75	0.77	0.00	0.72	0.72	0.72	0.70	0.00
RFor	0.84	0.84	0.84	0.83	0.30	0.84	0.84	0.84	0.78	0.33
Ridge	0.64	0.62	0.60	0.61	0.00	0.55	0.61	0.55	0.61	0.00
RCV	0.64	0.62	0.60	0.61	0.00	0.55	0.61	0.55	0.61	0.00
SGD	0.60	0.58	0.56	0.50	0.03	0.64	0.61	0.59	0.59	0.03
PAg	0.56	0.44	0.46	0.33	0.01	0.54	0.52	0.52	0.47	0.01
Bagg	0.81	0.80	0.81	0.82	0.21	0.77	0.77	0.77	0.76	0.21

**Table 9:** Building damage dataset ANOVA test analysis.

Features	sum_sq	df	F	PR(>F)
count_floors_pre_eq	79.395866	1	58.48394	2.456966 × 10^−14^
count_floors_post_eq	51.120105	1	37.49426	9.893068 × 10^−10^
age_building	20.806387	1	15.190723	0.000098
plinth_area_sq_ft	0.612941	1	0.446148	0.504202
height_ft_pre_eq	50.936849	1	37.358812	1.059888 × 10^−09^
height_ft_post_eq	30.589357	1	22.366279	0.000002
land_surface_condition	458.176972	1	358.153878	4.084559 × 10^−77^
foundation_type	7.723360	1	5.627706	0.017718
roof_type	45.032464	1	347.141642	7.018501 × 10^−75^
ground_floor_type	161.719556	1	120.636637	9.735479 × 10^−28^
floor_type	148.427174	1	110.494564	1.437504 × 10^−25^
position	9.226230	1	6.72431	0.09539
plan_configuration	97.724466	1	72.18645	2.575076 × 10^−17^
has_superstructure_adobe_1	6.160766	1	4.48805	0.034183
has_superstructure_1_mortar_stone	112.342837	1	83.170282	1.081052 × 10^−19^
has_superstructure_stone_flag	0.155581	1	0.113237	0.736503
has_super_cement_mortar_stone	1.970106	1	1.434296	0.231123
has_superstructure_1_mortar_brick	0.786869	1	0.572762	0.4492
has_supers_cement_mortar_brick	13.807720	1	0.765897	0.001516
has_superstructure_5	93.585132	1	69.085173	1.212492 × 10^−16^
has_superstructure_bamboo	59.854698	1	43.958894	3.719995 × 10^−11^
has_super_4_non_engineered	4.946706	1	3.602962	0.077736
has_superstructure_4_engineered	5.688974	1	4.567896	0.098789
has_superstructure_3	17.786486	1	12.979981	0.000318
condition_post_eq	308.967456	1	235.832275	5.282171 × 10^−52^
technical_solution_proposed	806.067947	1	667.625148	5.158686 × 10^−138^

**Table 10:** Damage grade PCA ANOVA classifier performance indices 80:20 before and after scaling.

Classifier	Before Feature Scaling					After Feature Scaling				
	Precision	Recall	F-Score	Accuracy	Runtime	Precision	Recall	F-Score	Accuracy	Runtime
LReg	0.68	0.65	0.63	0.68	0.14	0.67	0.64	0.64	0.67	0.06
KNN	0.41	0.40	0.40	0.57	0.06	0.74	0.73	0.74	0.77	0.12
KSVM	0.37	0.41	0.37	0.68	0.84	0.80	0.76	0.75	0.80	0.39
GNB	0.67	0.66	0.67	0.63	0.00	0.52	0.40	0.41	0.51	0.00
Dtree	0.79	0.78	0.79	0.81	0.07	0.72	0.72	0.72	0.75	0.07
Etree	0.77	0.77	0.77	0.78	0.00	0.70	0.69	0.70	0.71	0.01
RFor	0.86	0.86	0.86	0.82	0.32	0.86	0.86	0.86	0.79	0.35
Ridge	0.58	0.61	0.56	0.62	0.00	0.52	0.59	0.55	0.64	0.00
RCV	0.55	0.61	0.56	0.62	0.00	0.52	0.59	0.55	0.64	0.01
SGD	0.50	0.50	0.48	0.59	0.09	0.59	0.57	0.55	0.63	0.09
PAg	0.42	0.35	0.35	0.45	0.03	0.53	0.34	0.35	0.50	0.02
Bagg	0.82	0.82	0.82	0.83	0.26	0.74	0.74	0.74	0.79	0.26

**Table 11:** Damage grade PCA ANOVA classifier performance indices 70:30 before and after scaling.

Classifier	Before Feature Scaling					After Feature Scaling				
	Precision	Recall	F-Score	Accuracy	Runtime	Precision	Recall	F-Score	Accuracy	Runtime
LReg	0.64	0.62	0.59	0.66	0.14	0.66	0.64	0.63	0.67	0.06
KNN	0.41	0.40	0.40	0.52	0.06	0.74	0.73	0.74	0.76	0.13
KSVM	0.35	0.39	0.34	0.66	0.60	0.79	0.76	0.75	0.80	0.33
GNB	0.64	0.64	0.64	0.64	0.00	0.51	0.40	0.41	0.50	0.00
Dtree	0.77	0.77	0.77	0.55	0.07	0.71	0.71	0.71	0.76	0.07
Etree	0.76	0.75	0.76	0.80	0.00	0.70	0.69	0.70	0.73	0.00
RFor	0.88	0.87	0.88	0.82	0.27	0.88	0.87	0.88	0.79	0.29
Ridge	0.66	0.62	0.59	0.62	0.00	0.52	0.59	0.55	0.63	0.00
RCV	0.66	0.62	0.59	0.62	0.00	0.52	0.59	0.55	0.63	0.00
SGD	0.57	0.54	0.55	0.51	0.05	0.60	0.60	0.60	0.59	0.06
PAg	0.47	0.46	0.45	0.48	0.01	0.48	0.46	0.46	0.45	0.02
Bagg	0.81	0.81	0.81	0.82	0.25	0.76	0.76	0.76	0.78	0.23

**Table 12:** Damage grade PCA ANOVA classifier performance indices 60:40 before and after scaling.

Classifier	Before Feature Scaling					After Feature Scaling				
	Precision	Recall	F-Score	Accuracy	Runtime	Precision	Recall	F-Score	Accuracy	Runtime
LReg	0.65	0.64	0.63	0.67	0.14	0.69	0.67	0.66	0.67	0.04
KNN	0.41	0.39	0.39	0.53	0.08	0.76	0.76	0.76	0.77	0.14
KSVM	0.33	0.36	0.31	0.67	0.47	0.80	0.78	0.77	0.80	0.25
GNB	0.65	0.65	0.64	0.57	0.00	0.52	0.46	0.44	0.62	0.00
Dtree	0.79	0.79	0.79	0.80	0.06	0.73	0.73	0.73	0.77	0.05
Etree	0.75	0.75	0.75	0.77	0.00	0.72	0.72	0.72	0.76	0.00
RFor	0.86	0.86	0.86	0.82	0.30	0.86	0.86	0.86	0.79	0.33
Ridge	0.64	0.62	0.60	0.62	0.00	0.55	0.61	0.55	0.61	0.00
RCV	0.64	0.62	0.60	0.62	0.00	0.55	0.61	0.55	0.61	0.00
SGD	0.60	0.58	0.56	0.63	0.03	0.64	0.61	0.59	0.60	0.03
PAg	0.56	0.44	0.46	0.45	0.01	0.54	0.52	0.52	0.52	0.01
Bagg	0.81	0.80	0.81	0.81	0.21	0.77	0.77	0.77	0.79	0.21

**Table 13:** Performance analysis of ASR-DFCNN with existing classifiers.

Model	Accuracy	R-Squared Value
Kernel SVM	0.36	0.38
Gnaive Bayes	0.65	0.67
Decision tree	0.78	0.79
Extra tree	0.75	0.78
Random Forest	0.88	0.89
Bagging	0.80	0.82
DFCNN	0.87	0.86
ASR-DFCNN	0.98	0.97

## Data Availability

No new data was created in this study.
